# The influence of silicon on growth, drought stress tolerance and defense against *Neopestalotiopsis lusitanica*, in *Eucalyptus globulus*

**DOI:** 10.3389/fpls.2026.1894505

**Published:** 2026-08-03

**Authors:** Filipa L. Barros, Telma Nunes, Catarina I. Gonçalves, Helena Bragança, Eugénio Diogo, Rui Azevedo, Agostinho Almeida, Ana Quintela, Paula Melo

**Affiliations:** 1 Departamento de Biologia, Faculdade de Ciências, Universidade do Porto, Porto, Portugal; 2 INOV4Agro - GreenUPorto - Centro de Investigação em Produção Agroalimentar Sustentável & Departamento de Biologia, Faculdade de Ciências, Universidade do Porto, Porto, Portugal; 3 Instituto de Investigação da Floresta e Papel (RAIZ), Quinta de S. Francisco, Eixo, Portugal; 4 Instituto Nacional de Investigação Agrária e Veterinária, IP (INIAV, IP), Quinta do Marquês, Oeiras, Portugal; 5 BioISI, Biosystems & Integrative Sciences Institute, Faculty of Sciences, University of Lisbon, Lisboa, Portugal; 6 GREEN-IT Bioresources for Sustainability, ITQB NOVA, Oeiras, Portugal; 7 LAQV REQUIMTE, Faculdade de Farmácia, Universidade do Porto, Porto, Portugal

**Keywords:** biotic stress tolerance, calcium silicate, drought stress tolerance, nursery management, silicon absorption

## Abstract

Silicon (Si) is recognized as a beneficial element that enhances plant growth and stress tolerance in various species, although its effects in hardwoods remain unclear. This study evaluated the impact of Si supplementation on *Eucalyptus globulus* growth and its responses to drought and fungal infection. Two *E. globulus* genotypes were treated with 1 or 2 mM calcium silicate (CaSiO_3_), via substrate irrigation under nursery conditions, and Si content, plant growth, and productivity-related indicators were evaluated. After Si treatment, plants were subjected to drought stress using PEG-6000 or inoculated with *Neopestalotiopsis lusitanica.* In the drought-stressed plants, growth, stress markers, and antioxidant enzyme activities were evaluated. In the pathogen-inoculated, disease severity was assessed using mortality, lesion size, and symptom development. We observed that in genotype B, treatment with 2 mM CaSiO_3_ increased plant height by 60% and also increased photosynthetic pigments and starch content. In genotype A, the same treatment was particularly effective under stress conditions, increasing plant growth under drought stress by 50% and also reducing oxidative stress (decreasing proline, GSH and lipid peroxidation), enhancing glutamate dehydrogenase activity, and mitigating fungal infection by delaying lesion progression, symptom development and lowering plant mortality by 37.5% at the end of the trial. The effects were supported by the detection of Si in the leaves of both genotypes. These findings suggest that Si can enhance *E. globulus* growth and resilience and may represent a valuable tool in eucalyptus management, both in nurseries, where plants are often subjected to high-density cultivation and increased pathogen pressure, including emerging nursery-associated fungi such as *Neopestalotiopsis* sp, and under field conditions, where plants are frequently exposed to water limitations.

## 1 Introduction

Silicon (Si), the second most abundant element on Earth after oxygen, makes up about 27% of the crust and up to 70% of soil composition (Khan, 2025; Bakhat et al., 2018). It is classified as a beneficial element, Si supports certain plant species in specific environments as it may aid in balancing excess elements, limiting uptake of harmful nutrients, or substitute for essential nutrients when absent (Khan, 2025; Bakhat et al., 2018; Frew et al., 2018). Si readily combines with oxygen to form insoluble silicates such as quartz, feldspar and clay minerals. Consequently, only a small fraction is present in soil solution as soluble orthosilicic acid (H_4_SiO_4_), the form that can be absorbed by plant roots, and translocated through the transpiration stream (Khan, 2025; Khan et al., 2023; Pandey et al., 2025). Silicon accumulation in plants is largely determined by phylogeny rather than environmental factors such as soil Si or pH (Guntzer et al., 2012; Bakhat et al., 2018). Accordingly, Si concentration varies widely among species, with generally higher levels reported in monocots, particularly within the Poacaea family (Guntzer et al., 2012; Luyckx et al., 2017). Plants can be further classified based on shoot Si concentrations into accumulators (100–150 g SiO_2_ kg^−1^), intermediate types (10–50 g SiO_2_ kg^−1^), and non-accumulators (below 5 g SiO_2_ kg^−1^), reflecting inherent differences in Si uptake and transport mechanisms among species (Broadley et al., 2012; Mateus et al., 2017).

Silicon (Si) plays a key role in promoting environmentally sustainable agriculture by enhancing plant growth and mitigating the negative effects of stress (Wang et al., 2017; Frew et al., 2018; Khan et al., 2023; Wang et al., 2021; Khan, 2025). Numerous studies have demonstrated its potential as a plant growth promoter across different crops, improving yield, biomass accumulation, photosynthetic performance, and product quality (Ahmed et al., 2008; Pilon et al., 2013; White et al., 2017; Shwethakumari and Prakash, 2018; Artyszak, 2018). In addition, Si can influence carbon-to-nitrogen balance and nutrient use efficiency, supporting plant metabolism during key developmental stages (Luyckx et al., 2017). Various application methods have been explored, including fertigation, soil uptake, and foliar spraying (Artyszak, 2018; Khan et al., 2023). Under stress conditions, Si contributes to the formation of chemical and physical barriers that mitigate abiotic and biotic stresses, strengthening agricultural resilience in a changing climate (Wang et al., 2017; Bakhat et al., 2018; Wang et al., 2021; Khan et al., 2023).

In particular, drought stress is one of the most critical environmental factors limiting plant growth and productivity, as it directly affects physiological processes such as photosynthesis, nutrient uptake, and cellular stability (Wang et al., 2021; Du et al., 2024; Khan et al., 2025). To cope with drought conditions, plants respond through integrated physiological, biochemical, and molecular mechanisms, including osmotic adjustment, antioxidant systems, and stress-responsive signaling pathways (Basirat et al., 2019; Du et al., 2024; Khan et al., 2025; Parmar et al., 2025). Silicon (Si)-mediated drought stress resistance has been shown to involve reduced transpiration (through the formation of a silica cuticle and reduced guard cell turgor), enhanced water use efficiency, root elongation and improved water absorption (via increased aquaporin gene expression). Additionally, Si plays a crucial role in plant antioxidant metabolism, including the enhancement of the activity of antioxidant defense enzymes, such as peroxidase, catalase and superoxide dismutase, and increasing the levels of non-enzymatic metabolites like polyamines and reduced glutathione (Shi et al., 2014; Frew et al., 2018; Malik et al., 2021; Ranjan et al., 2021; Song et al., 2021; Wang et al., 2021; Cheraghi et al., 2023; Nascimento-Silva, 2023; Cheraghi et al., 2024).

Silicon has also been implicated in protection against biotic stress. This element provides a natural defense system against various insects and pathogens by strengthening plant tissues, inducing the production of defense compounds, and activating systemic defense responses (Bakhat et al., 2018; Ranjan et al., 2021; Song et al., 2021; Khan et al., 2023; Tenguri et al., 2023). In addition, Si modulates lignin synthesis and increases the levels of several antimicrobial compounds, such as flavonoids, phytoalexins, pathogenesis-related proteins, and phenolic compounds (Broadley et al., 2012; Wang et al., 2017; Bakhat et al., 2018;). Furthermore, Si upregulates genes related to pest and disease resistance and influences phytohormones such as ethylene, salicylic acid, and jasmonic acid (Wang et al., 2017; Frew et al., 2018; Song et al., 2021; Khan et al., 2023; Tenguri et al., 2023).

Although numerous studies have been conducted on Si behavior in plants, hardwood species have received comparatively little attention, despite their widespread distribution and economic importance. Native to Australia, the genus *Eucalyptus* is now found in various climates worldwide (Belisário et al., 2020; Tomé et al., 2021), covering 18 million hectares across 90 countries, and ranking among the most valuable and widely cultivated hardwood trees genera (Pirralho et al., 2014). Its physical and chemical properties make it economically important in industries such as wood, fuelwood, lumber, pulp, pharmaceuticals, fragrances, and extractive sectors (Belisário et al., 2020). *Eucalyptus globulus* was the first species to gain global recognition and is primarily used for mine supports, firewood, and pulpwood production (FAO, 1978, 2001). A significant challenge in eucalyptus plantations is water depletion, particularly as drought conditions intensify due to global warming and climate change (Saadaoui et al., 2017; Yang et al., 2023). Drought stress is especially challenging in the nursery to field transitions, as the growing conditions can change substantially (Correia et al., 2014; Tomé et al., 2021).

As an introduced (exotic) species in the Mediterranean region, *E. globulus* has faced relatively few diseases (Bragança et al., 2016; Diogo et al., 2021). Over the past decades, this scenario has changed, with fungal diseases such as leaf spot, gray mold, rust, cutting rot, and damping-off becoming major challenges to eucalyptus growth (Belisário et al., 2020; Diogo et al., 2021). Specifically, in Portugal, there has been a notable increase in the incidence of fungal diseases affecting eucalyptus nurseries, sometimes associated with intense mortality during the early stages of plant production, as documented in several studies (Silva et al., 2012, 2014; Barradas et al., 2016; Diogo et al., 2021). *Neopestalotiopsis* is a fungus generally regarded as a secondary pathogen that affects several economically important plant species (Gaire et al., 2026). Recent research by Diogo et al. (2021) identified five new species (formerly classified as *Pestalotiopsis*) and recognized them as primary pathogen of *E. globulus*. These species are associated with a disease characterizedby leaf necrosis, stem girdling, and dieback, occurring mainly in nurseries, forest yards where young eucalypts await plantation, and shortly after plantation (within a few months), with mortality ranging from a few plants to 30–40% (Belisário et al., 2020; Santos et al., 2020; Diogo et al., 2021). The disease tends to cause more severe damage in physiologically stressed cuttings or in plants with injuries resulting from vegetative propagation (Maharachchikumbura et al., 2014; Belisário et al., 2020). Despite its impact, specific studies on this disease remain limited (Belisário et al., 2020; Santos et al., 2020).

This study aimed to evaluate the potential of incorporating Si into the fertilization of *E. globulus* to support the transition from nursery conditions to field plantations, where plants are exposed to multiple stressors, including drought and pests. Although the benefits of Si accumulation have been widely documented in several plant species, its role in *E. globulus* remains poorly understood, with few studies addressing the use of silicate-based fertilizers. In addition, genotypic variation influences Si uptake within species (Wu et al., 2006; Ma et al., 2007), emphasizing the importance of the genetic background in silicon acquisition. To address these knowledge gaps, we investigated the effects of Si application, supplied as calcium silicate via soil irrigation, on growth and performance *E. globulus* under non-stress conditions and under water deficit and infection by *Neopestalotiopsis lusitanica*. Furthermore, we compared the responses of two *E. globulus* genotypes with differing susceptibilities to fungal infection, aiming to elucidate the interaction between Si supplementation and genotype-dependent stress tolerance.

## 2 Materials and methods

### 2.1 Materials and growth conditions

Three-month-old, well-established *E. globulus* plants were used in this study, as they retain a high degree of physiological plasticity while being sufficiently developed to avoid stem breakage during handling. Two genotypes differing in their susceptibility to fungal infection were selected based on previous observations from trials conducted by RAIZ and INIAV, genotype A (clone 102051011, high susceptibility) and genotype B (clone 100074002, moderate susceptibility), obtained by rooted cutting (Viveiros Aliança - Empresa Produtora de Plantas, S.A). Plants were supplied to the RAIZ facilities (Eixo, Portugal) in May 2024. and were grown in nursery pots with a turf-perlite substrate (Siro, Portugal) supplemented with a controlled-release coated fertilizer (12-7–18 NPK w/w, with micronutrients Osmocote bloom, ICL Specialty Fertilizers) with a release period of 2–3 months. Upon arrival, the plants received an additional supplementation with 100 ppm ammonium sulphate and irrigated as required (typically once or twice per week) with water from an artisanal well (composition listed in Online Resource 1).

The reagents used in this study were calcium silicate (purum grade, 12-22% Ca as CaO, ≥87% SiO_2_, Sigma-Aldrich), calcium chloride dihydrate (Merck), ammonium sulphate (99.5%, Merck), and polyethylene glycol 6,000 (PEG-6000, Alfa Aesar). All solutions were prepared using water from the same source and adjusted to pH 5.5-6.0. Irrigation treatments consisted of water as the control, calcium chloride (2 mM) as the element control, and calcium silicate at 1 mM or 2 mM. These concentrations were selected based on previous studies (Mateus et al., 2017; Queiroz et al., 2018). Plants were maintained under greenhouse conditions from May to July 2024, with temperatures ranging from 20 °C to 35 °C, reflecting spring and summer conditions. Throughout the experiments, the eucalyptus plants were rearranged in a randomized complete block design (RCBD).

### 2.2 Growth experiment

After one week of acclimatization at RAIZ’s (Forest and Paper Research Institute) greenhouse (May 2024), the plants from both genotypes (A and B) received Si treatments applied twice per week for 5 weeks, with no additional nutrient supplementation. In total, 50 plants of each genotype were prepared per treatment. By the end of this period, plant height differences (differences in height between the beginning and the end of the growth experiment) were evaluated, and leaves were collected, pooled on 3–5 plant samples, ground in liquid nitrogen and stored at -80 °C for biochemical quantification of the physiological effects of Si treatment.

### 2.3 Drought stress experiment

By the end of the 5-week growth assay (June 2024), a subset of 10–15 plants of genotype A were irrigated with 5% PEG-6000 (Dasgupta and Dharanishanthi, 2017), once a week for four weeks, and a control subset of 10–15 plants continued to be watered. After this period, plant heights (differences between the beginning and the end of the drought stress experiment) were evaluated, and leaves were collected and stored at -80 °C (as previously described) for biochemical quantifications of the physiological effects of Si treatment under drought stress conditions.

### 2.4 Fungal infection experiment

Following the initial 5-week period with Si fertilization (June 2024), a subset of 10–15 plants of each genotype (with different susceptibilities to fungal infection, with genotype A being the most susceptible) was transferred to a climatic chamber (21 °C, 70% relative humidity and 16:9 L:D photoperiod, with lighting provided by five Cool White 840 fluorescent lamps, positioned around 50 cm away from the plants. Ten plants were inoculated with spores from *Neopestalotiopsis lusitanica* (strain MEAN – 1318) (MEAN collection – Instituto Nacional de Investigação Agrária e Veterinária I.P. -INIAV I.P.). The inoculation process was developed in collaboration with INIAV and RAIZ and involved the introduction of fungal spores through three punctures made with a syringe needle (0.5 mm in diameter). Prior to inoculation, the needle was used to gently scrape the surface of a 15-day-old potato dextrose agar (PDA) culture showing abundant sporulation, covering approximately 3 cm of the culture surface. The punctures were performed in the basal portion of the stem, approximately 2–3 cm above the mother stake, with each puncture spaced about 2 cm apart, avoiding any pre-existing lesion (**Supplementary Figure 1**)s. Control plants were not inoculated but punctured with a sterile needle following the same procedure. The plants were evaluated every 3 days for symptom severity caused by *N. lusitanica* (plant health), necrotic lesion size (expressed as a mean percentage of lesion size relative to plant height) and mortality rate (percentage of dead plants). The surveys were finalized when the genotypes exhibited near-total mortality and/or when the lesion size ceased to increase. Plant health was recorded according to five categories (with representative images for each category provided in the **Supplementary Material**, **Supplementary Figure 2**): Level 1 – Necrotic lesions are present on the stem, but the plant shows no visible signs of physiological decline; Level 2 – Initial leaf desiccation is observed, though the plant remains overall healthy; Level 3 – Foliar desiccation is evident, especially at the apex, but the plant is still viable; Level 4 – Widespread desiccation and prominent necrotic lesions are present; the plant is severely affected and approaching death; Level 5 – The plant is fully desiccated and/or dead.

### 2.5 Evaluation of Si content

The leaves of 4–5 plants from each genotype and each Si treatment were washed in distilled water, air-dried at room temperature (RT) for 24 to 48 hours, frozen at -80 °C and lyophilized in a BenchTop Pro vacuum freeze dryer (SP Scientific, Warminster, USA) at 65°C until constant mass and then smashed in a mortar. Samples were mineralized by closed-vessel microwave-assisted acid digestion in an ETHOS™ EASY microwave oven (Milestone, Sorisole, Italy) equipped with an SK-15 EasyTEMP high-pressure rotor, following a procedure based on a U.S. Environmental Protection Agency (EPA) method. Briefly, ~0.4 g of sample, 7 mL of high-purity nitric acid (HNO3 ≥ 69%, TraceSELECT™, Honeywell Fluka, Seelze, Germany), 0.5 mL of high-purity hydrofluoric acid (HF 47–51%, TraceSELECT™, Fluka), and 1 mL of high-purity hydrogen peroxide (H2O2 30%, Suprapur^®^, Supelco, Darmstadt, Germany) were added to the vessels and placed in the microwave oven (20 min ramp up to 210 °C, followed by 15 min at 210 °C). After cooling to room temperature, the vessels were opened and 5 mL of 4% (m/v) H3BO3 (>99.5%, Sigma-Aldrich, St. Louis, MO) was added, and the vessel place inside the microwave oven again (20 min ramp-up to 160° C, followed by 10 min at 160 °C). Finally, sample solutions were transferred to 50 mL tubes, and the volume was adjusted with ultrapure water (resistivity >18.2 MΩ.cm at 25 °C; Arium^®^ pro, Sartorius, Göttingen, Germany) to 30 mL. Sample blanks were obtained using the same procedure. Each sample was digested in triplicate. For analytical quality control, a certified reference material BCR-129 (Joint Research Centre, European Commission, Geel, Belgium) was mineralized and analyzed as the samples.

The analysis of the solutions was performed by inductively coupled plasma – mass spectrometry (ICP-MS) with an iCAP™ Q (Thermo Fisher Scientific, Waltham, MA, USA) instrument. Prior to the analytical run, the instrument was tuned for maximum sensitivity and signal stability, and for minimal formation of oxides and double-charge ions.

Sample solutions were diluted 1:10 with a solution containing 2% v/v HNO3 and 10 µg/L internal standard (IS; Internal Standard Mix 1 - SCP-IS7, 10 mg/L, PlasmaCAL^®^, SCP Science, Quebec, Canada). The elemental isotope 28Si was measured for analytical determination, and the elemental isotope 6Li was monitored as IS.

### 2.6 Extraction and quantification of photosynthetic pigments, total soluble sugars and starch content

Pigment concentrations (Chl *a*, Chl *b*, and Car) were calculated according to Lichtenthaler and Buschmann (2001). Fifty mg of plant material was macerated with quartz sand in 5 mL of 96% ethanol and centrifuged at 5,000× *g* for 10 minutes. Absorbance at 647, 663, and 470 nm was measured (Multiskan GO, Thermo Scientific, Massachusetts, United States) using ethanol 96% as the blank. For the quantification of soluble sugar content, the supernatant (SN) from pigment quantification was adjusted to 80% ethanol, and the quantification was performed as described in Roque et al. (2023). Starch quantification was determined in the pellet of the previous centrifugation, according to Roque et al. (2023).

### 2.7 Quantification of proline, glutathione, H_2_O_2_ and lipid peroxidation

For proline (Pro) and glutathione (GSH) quantification, 200 mg of plant material was homogenized in 3% (w/v) sulfosalicylic acid. Pro was detected with acidic ninhydrin, after extraction with toluene, and GSH using Ellman`s Reagent [DTNB: 5,5-dithiobis-(2-nitrobenzoic acid)], as previously described in Brito et al. (2022). H_2_O_2_ and malondialdehyde (MDA-indicative of lipid peroxidation) contents were quantified, as described in Brito et al. (2022), in 200 mg of plant material homogenized in 0.1% (w/v) trichloroacetic acid (TCA). The contents in H_2_O_2_ and MDA were determined spectrophotometrically by reaction with potassium iodide and with 0.5% (w/v) thiobarbituric acid (TBA) in 20% (w/v) TCA, respectively.

### 2.8 Enzyme extraction and quantification of activity

Soluble protein content was quantified using the Bradford (1976) method. Extracts from enzymatic buffers were mixed with Coomassie reagent (Bradford Dye Reagent, Alfa Aesar), incubated in the dark for 20 minutes, and absorbance was measured at 595 nm. Protein content was determined using a serum albumin protein (BSA) standard curve. Catalase (CAT) and ascorbate peroxidase (APX) activity were measured following Brito et al. (2022). Guaiacol peroxidase (GPOX) activity was measured following Mariz-Ponte et al. (2018). Glutamate dehydrogenase (GDH) activity was measured following Ferreira et al. (2019).

### 2.9 Statistical analysis

For growth assessment, approximately 50 plants per genotype were used for each treatment. In the drought stress and *N. lusitanica* inoculation assays, 10–15 plants were used per treatment. Biochemical analyses were performed using 3–5 pooled samples, each with at least three independent technical replicates, and results were expressed as mean ± SD. Statistical analyses were selected according to dataset size. Comparisons between two groups were performed using Student’s *t*-test. For analyses involving more than two groups, one-way ANOVA was applied, followed by Sidak’s test for comparisons with a control and Tukey’s test for multiple comparisons. Statistical significance was set at *P* ≤ 0.05 (95% confidence level). Analyses were conducted using GraphPad Prism 8 software (v8.0.1).

## 3 Results

### 3.1 Effect of Si on *E. globulus* growth

**Figure 1:**
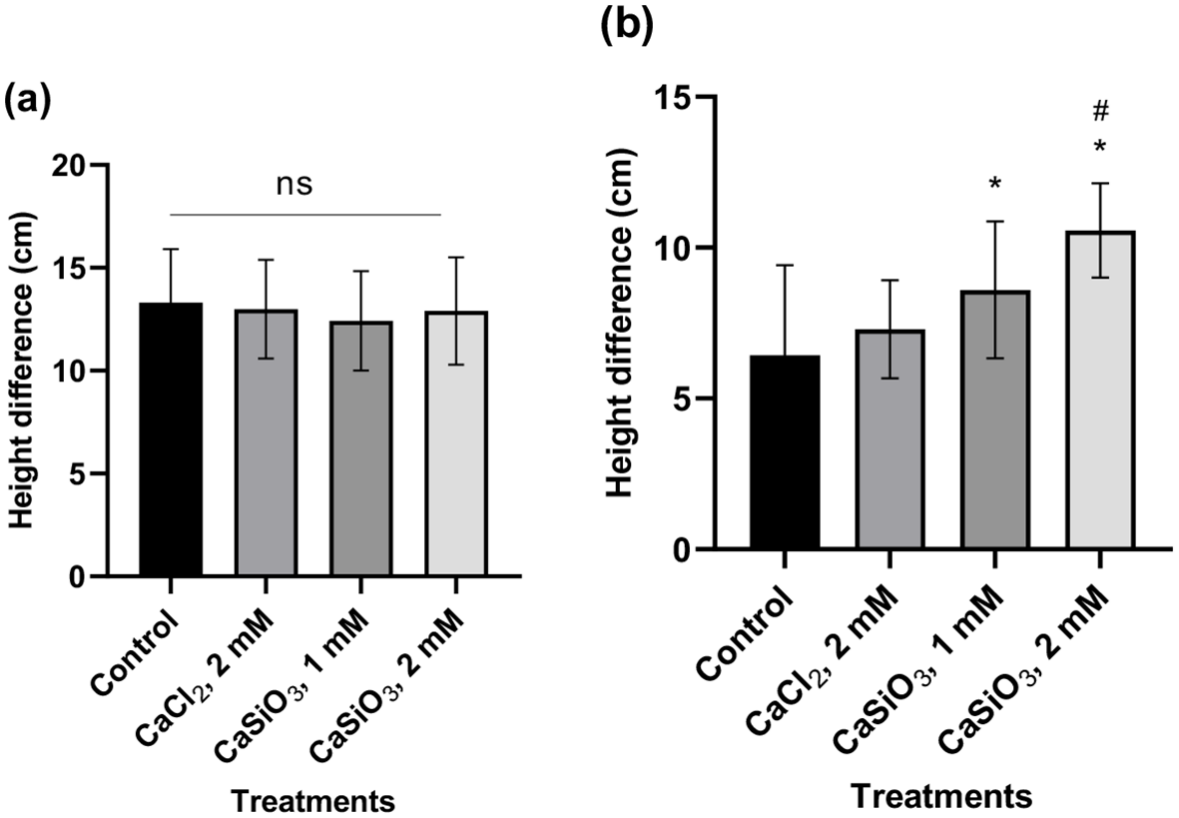
Effect of Si on *Eucalyptus globulus* growth. Plants of genotypes A **(a)** and B **(b)** were measured after a 5-week treatment, and differences in height relative to the beginning of the growth experiment were determined (Height differences). The results were expressed as means ± SD. Statistically significant differences compared to the control plants are represented with an asterisk, statistically significant differences compared to the element control CaCl_2_ are represented with a cardinal, and no statistical significances were indicated as ns (Sidak’s multiple comparison analysis, *P* < 0.05).

In this study, we investigated the application of Si in the form of calcium silicate (1 mM and 2 mM) via soil irrigation as part of *E. globulus* fertilization, aiming to evaluate the growth-promoting effects of Si, using two genotypes A and B. The differences in plant height between the beginning and the end of the experiment are represented in **Figure 1**. For genotype A, no significant differences in plant height were detected between Si-treated plants and control plants. In contrast, for genotype B, treatments with 1 and 2 mM of calcium silicate resulted in a significant increase in plant height. Moreover, plants treated with 2 mM calcium silicate exhibited a statistically significant increase in height compared with the calcium control (CaCl_2_), indicating that the observed growth-promoting effects can be attributed to silicon rather than calcium.

**Figure 2:**
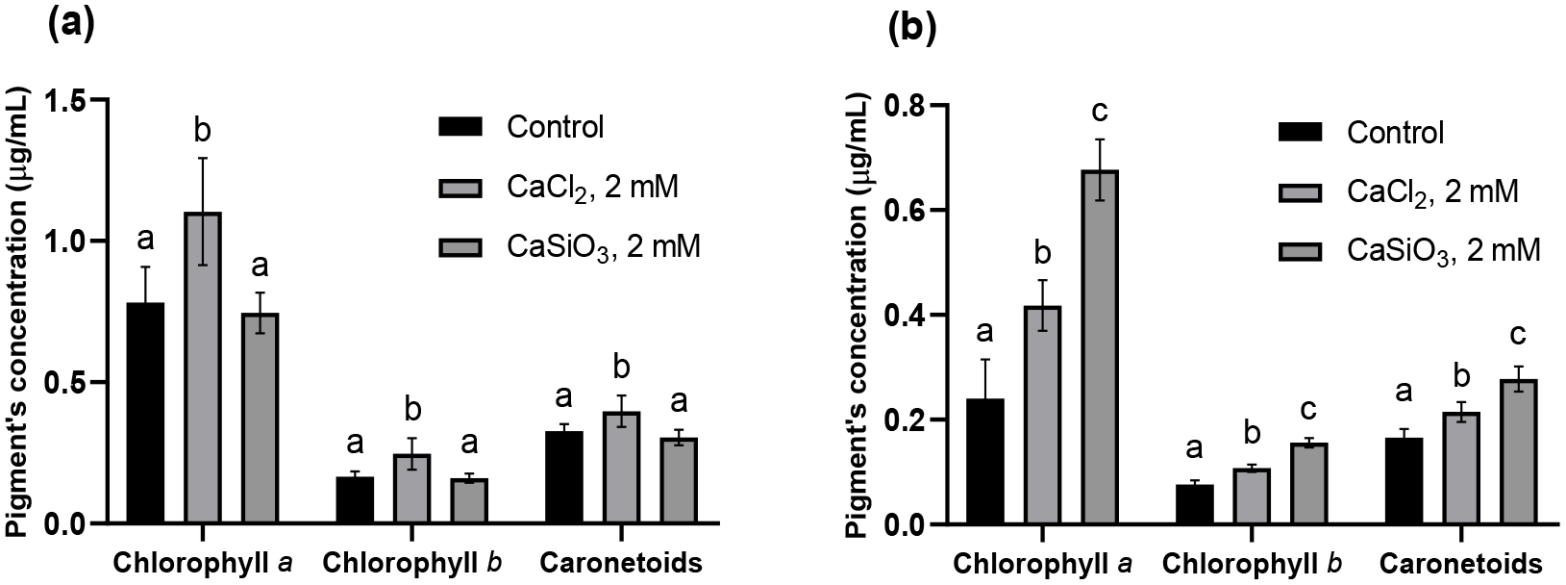
Concentration of photosynthetic pigments in plants of genotypes A **(a)** and B **(b)** after a 5-week treatment. The results were expressed as means ± SD. Statistically significant differences are represented with different letters (Tukey test, *P* < 0.05).

To gain a comprehensive understanding of the overall physiological conditions of the plants treated with Si, we assessed several key productivity-related indicators, such as photosynthetic pigments, total soluble sugars, and starch content, in the 2 mM calcium silicate treatment plants due to its promising growth effects. As observed in **Figure 2a**, genotype A plants treated with 2 mM calcium silicate showed no significant difference compared to the control. In genotype B (**Figure 2b**), treatment with 2 mM calcium silicate resulted in a significant increase in photosynthetic pigments compared to both the control and the element control.

**Figure 3:**
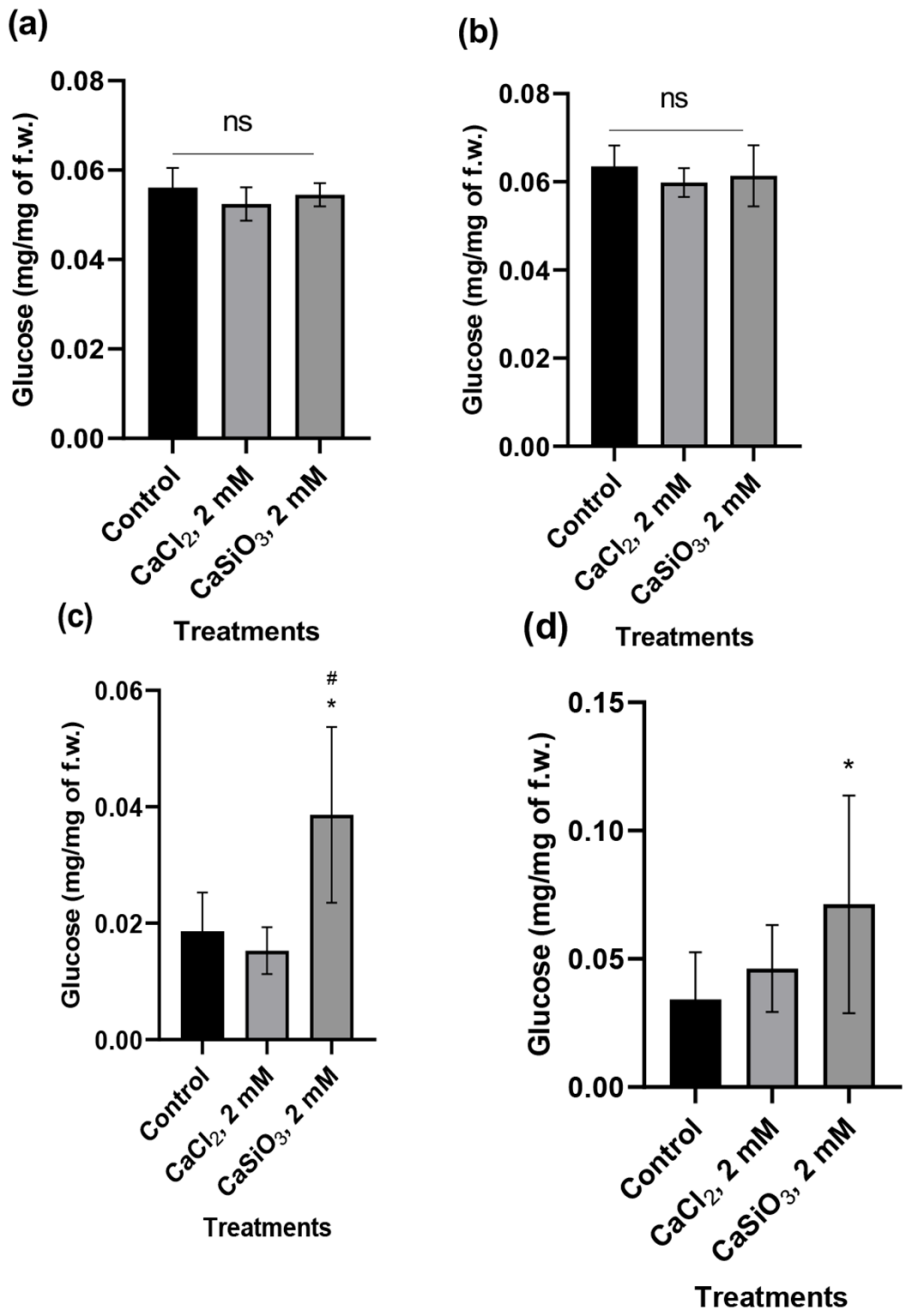
Concentration of the total soluble sugar **(a, b)** and starch (quantitatively converted into glucose) (**c, d**) content on genotypes A **(a, c)** and B **(b, d)** after a 5-week treatment. The results were expressed as means ± SD. Statistically significant differences compared to the control plants are represented with an asterisk, statistically significant differences compared to the element’s control are represented with a cardinal, and no statistical significances were indicated as ns (Sidak’s multiple comparison analysis, *P* < 0.05).

The sugar content of the plants of genotypes A and B did not differ significantly from the control, with all treatments averaging the same sugar level (**Figures 3a, b**). Regarding the starch content (**Figures 3c, d**), calcium silicate at 2 mM significantly increased starch content in both genotypes A and B compared to both water and element controls.

### 3.2 Silicon effect on drought stress tolerance

**Figure 4:**
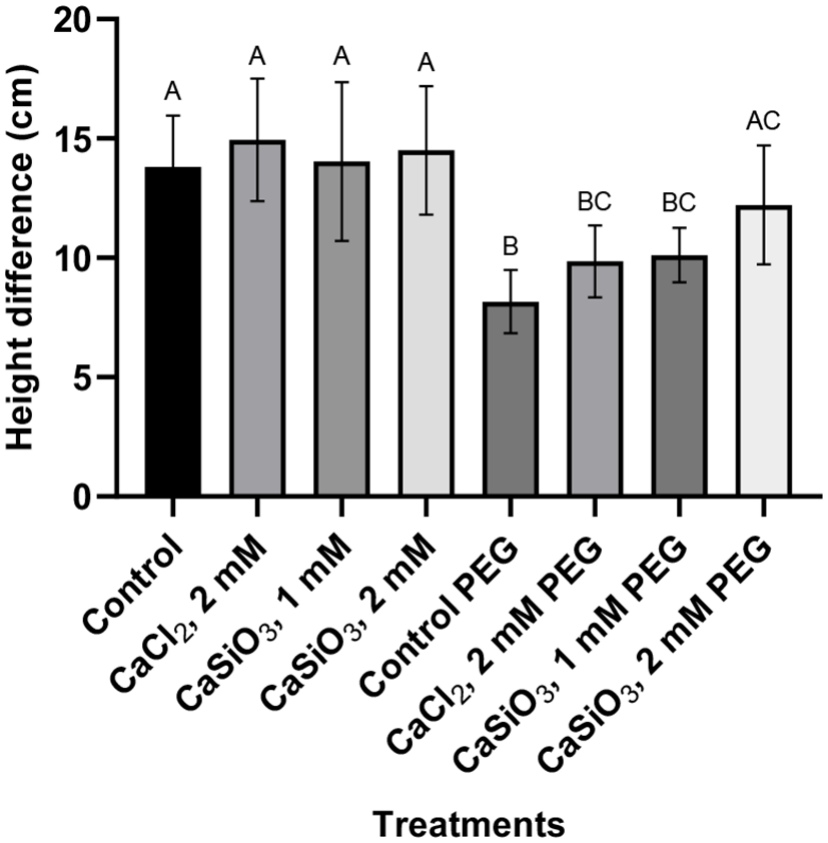
Effect of Si on the growth of *Eucalyptus globulus* under drought stress. Plants of genotype A were measured after a 4-week period of drought stress treatment, and differences in height relative to the beginning of the experiment were determined (Height differences). The results were expressed as means ± SD. Statistically significant differences within each Si form subset are represented with different letters (Tukey test, *P* < 0.05).

To evaluate the role of Si in alleviating drought stress, Si-treated plants of genotype A were watered with polyethylene glycol (PEG) solution to induce water deficit. Plant heights were measured at the beginning and after 4 weeks of treatment, and growth was calculated as the difference between these time points (**Figure 4**). Under non-stress conditions, no significant differences in growth were observed among treatments. In contrast, under PEG-induced stress, plants treated with 2 mM calcium silicate exhibited a significant increase in growth compared to the PEG-control and exceeded the growth observed in the PEG-element control. Additionally, no significant differences were detected between 2 mM Si-treated plants grown with or without PEG, suggesting that Si application mitigates the negative effects of drought stress.

**Figure 5:**
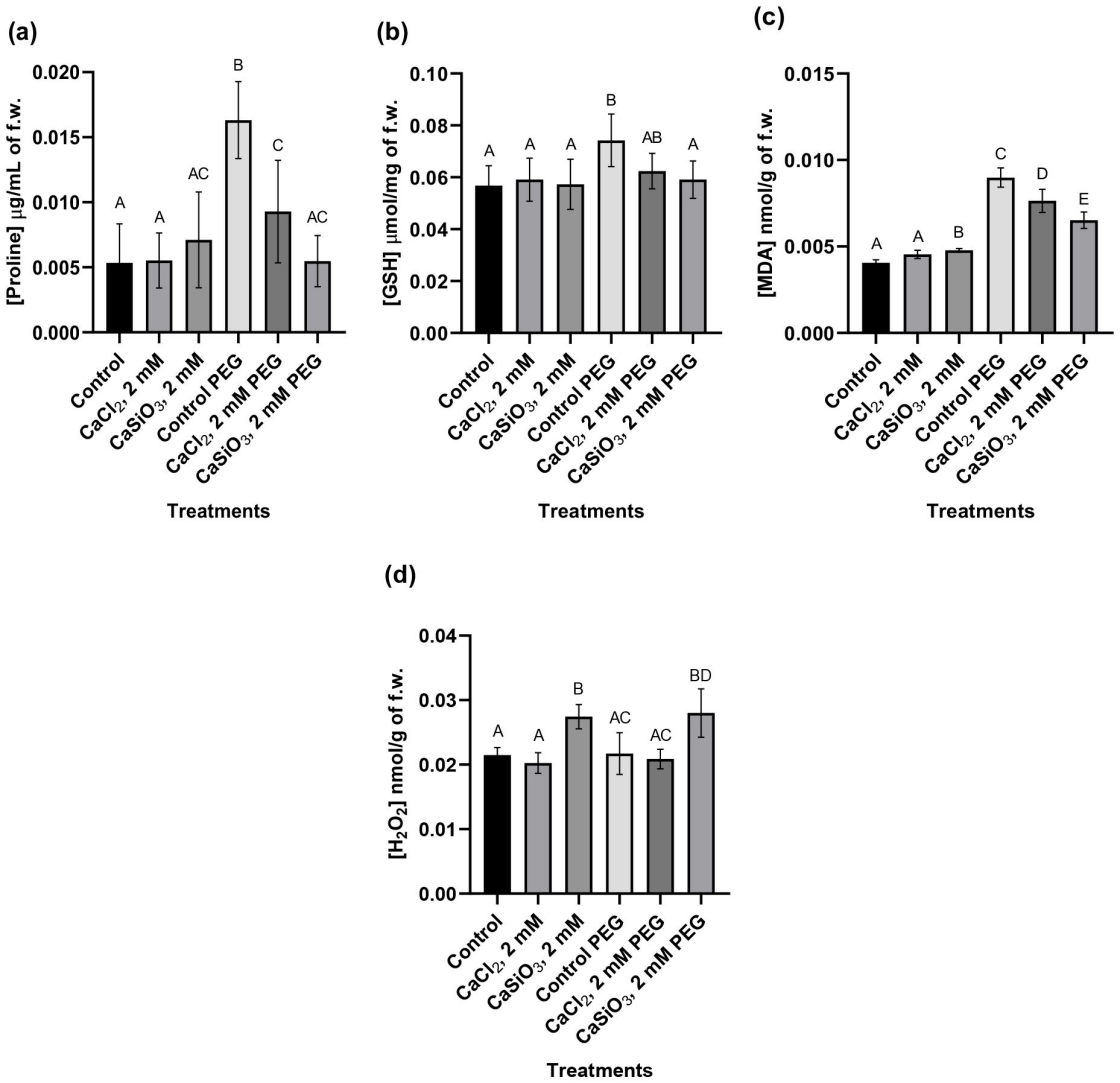
The influence of Si on Proline **(a)**, GSH **(b)**, MDA **(c)** and H_2_O_2_ **(d)** contents in the leaves of plants of genotype A. The results were expressed as means ± SD. Statistically significant differences are represented with different letters (Tukey test, *P* < 0.05).

To further evaluate the effect of Si on mitigating the drought stress response in *E. globulus*, several biochemical stress-related parameters were analyzed in plants treated with 2 mM calcium silicate, which showed the most significant effect in alleviating growth impairment under drought stress. Regarding Pro levels (**Figure 5a**), when comparing stressed control plants to their respective unstressed control, an expected increase in Pro levels was observed. Under drought stress, 2 mM calcium silicate demonstrated a statistically significant decrease in Pro levels compared with controls. Furthermore, this decrease was enough to restore Pro levels to normal, with no significant differences between the stressed 2 mM calcium silicate treatment and its unstressed counterparts. As for the GSH levels (**Figure 5b**), no significant differences were observed between unstressed plants. However, under drought stress, a significant increase in GSH was detected in control PEG plants. Furthermore, treatment with 2 mM calcium silicate reduced GSH levels to those of the corresponding non-stressed plants, suggesting that Si affects GSH levels under drought-stress conditions. Furthermore, a significant increase in MDA levels was observed in stressed plants, as predicted (**Figure 5c**). However, under drought stress, 2 mM calcium silicate treatment significantly decreased MDA levels compared to the PEG-treated controls, demonstrating a clear effect of Si in alleviating drought stress. As for H_2_O_2_ levels (**Figure 5d**), in both unstressed and drought-stressed conditions, calcium silicate significantly increased H_2_O_2_ levels compared to both controls.

**Figure 6:**
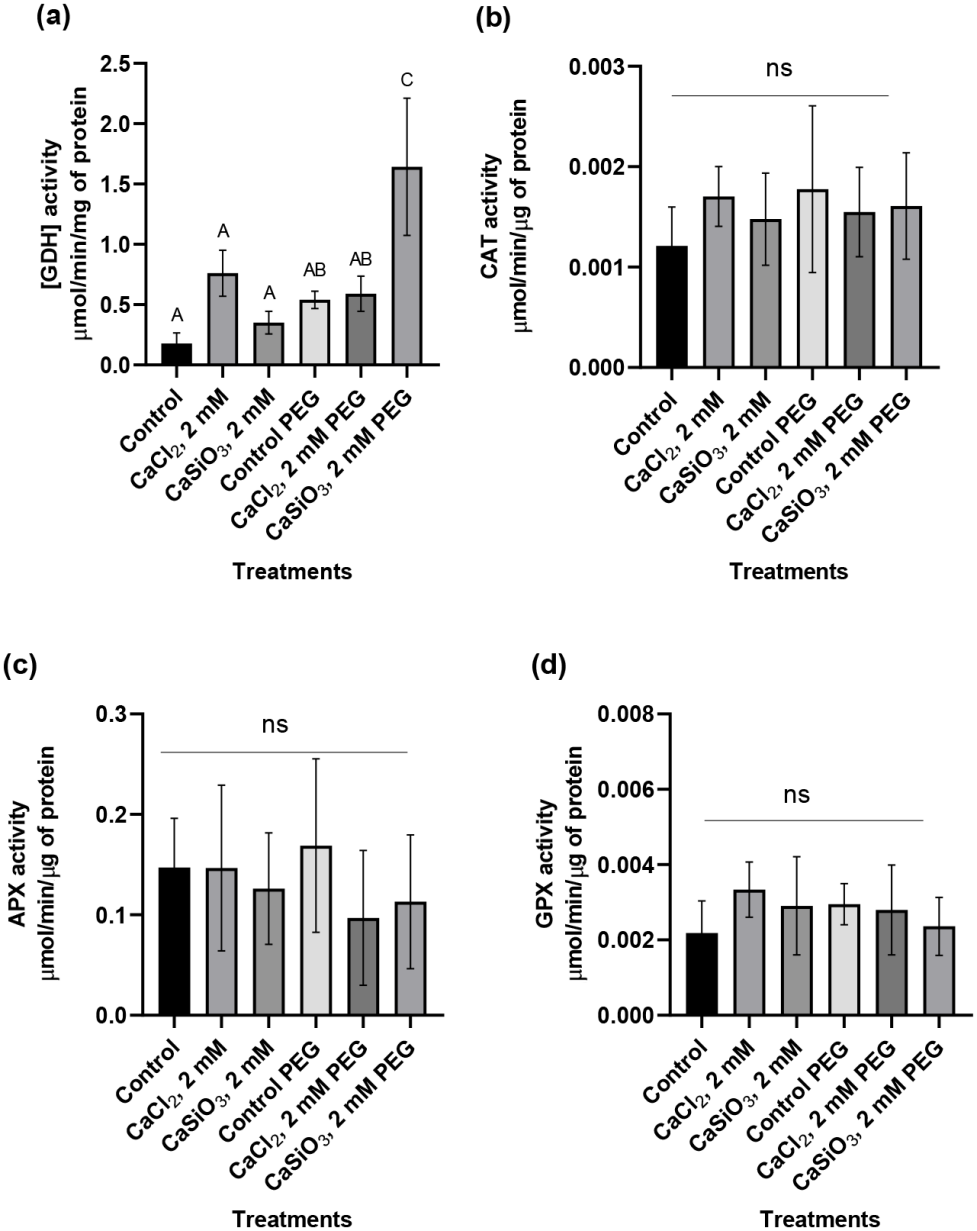
The influence of Si on GDH **(a)**, CAT **(b)**, APX **(c)** and GPOX **(d)** activity on the leaves of plants of genotype A. The results were expressed as means ± SD. Statistically significant differences are represented with different letters, and no statistical significances were indicated as ns (Tukey test, *P* < 0.05).

For GDH activity (**Figure 6a**), no significant differences were observed among unstressed plants. Although drought-stressed control plants tended to display higher GDH activity than unstressed control plants, these differences were not statistically significant. In contrast, drought-stressed plants treated with calcium silicate showed a significant increase in GDH activity compared to both unstressed plants and their respective unstressed controls, indicating a strong effect of Si under PEG-induced stress. In the case of CAT, APX and GPOX activity (**Figures 6b-d**), no significant differences were observed between unstressed and stressed plants or among treatments.

### 3.3 Silicon impact on eucalyptus resistance to *Neopestalotiopsis lusitanica*

**Figure 7:**
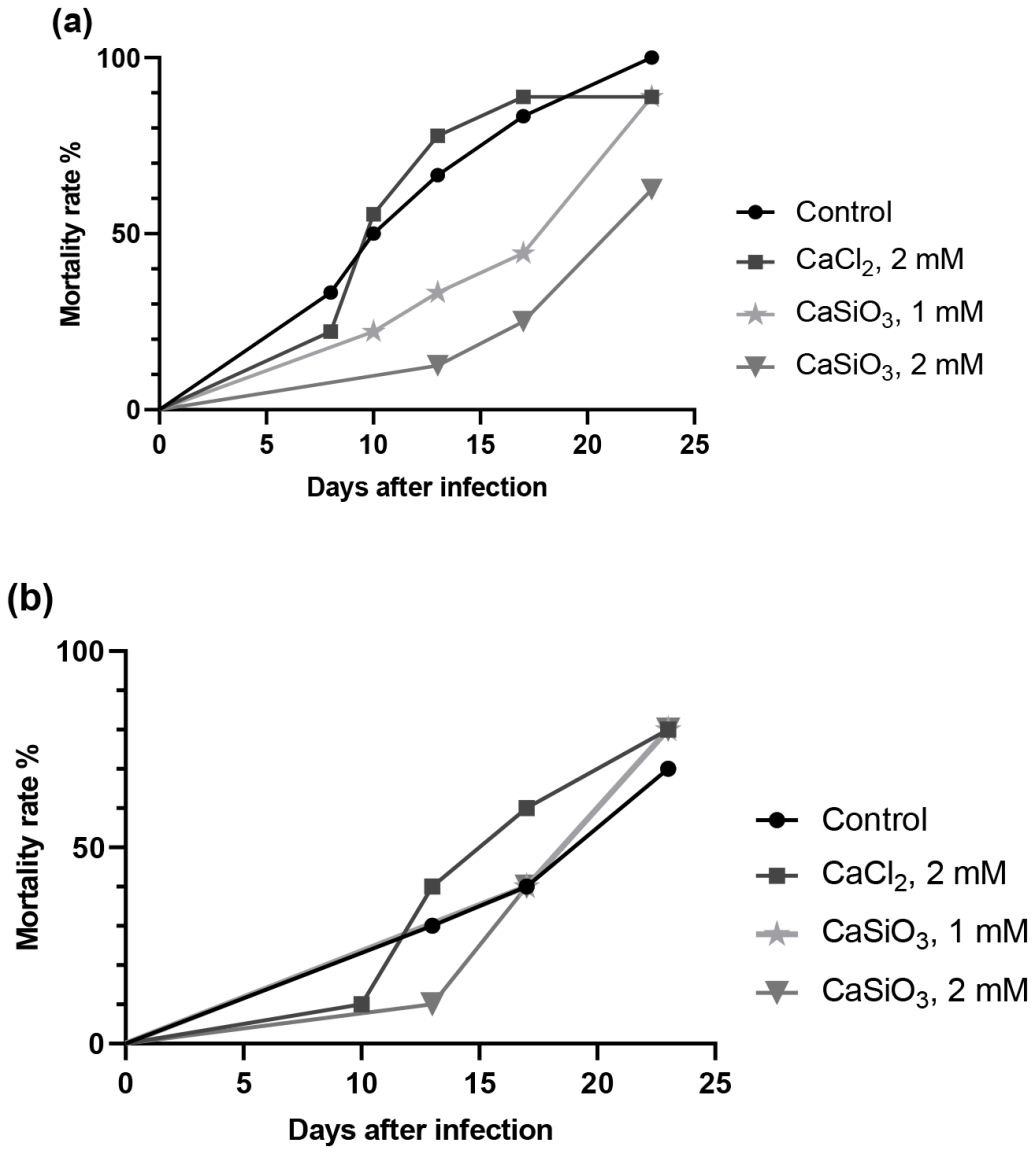
Mortality rate (%) of genotype A **(a)** and B **(b)** infected with *Neopestalotiopsis lusitanica* under different treatments during a 25-day monitoring period.

To evaluate the effects of Si on *E. globulus* resistance to biotic stress caused by *N. lusitanica*, two genotypes, A and B, with different susceptibility, were analyzed. In genotype A (**Figure 7a**), plant mortality rate began to increase at day 8. Throughout the experiment, treatment with 2 mM calcium silicate consistently resulted in the lowest rates. By day 23, the control reached 100%, whereas plants treated with 2 mM calcium silicate maintained the lowest mortality (50%), indicating a strong protective effect against *N. lusitanica* infection in genotype A. For genotype B (**Figure 7b**), no mortality was observed until day 8, consistent with its lower susceptibility to the pathogen. Mortality began to increase at day 10, with higher values observed in the element control. Between days 13 and 23, mortality progressively increased across all treatments, although plants treated with 2 mM calcium silicate consistently showed the lowest values. By day 23, mortality levels were substantial across all groups, with the control group showing the lowest rate at 70%, while calcium silicate treatments and 2 mM calcium chloride reached 80%. These results suggest that Si application may confer partial protection against *N. lusitanica* during the early stages of infection on genotype B; however, this protective effect appears to diminish over time.

**Figure 8:**
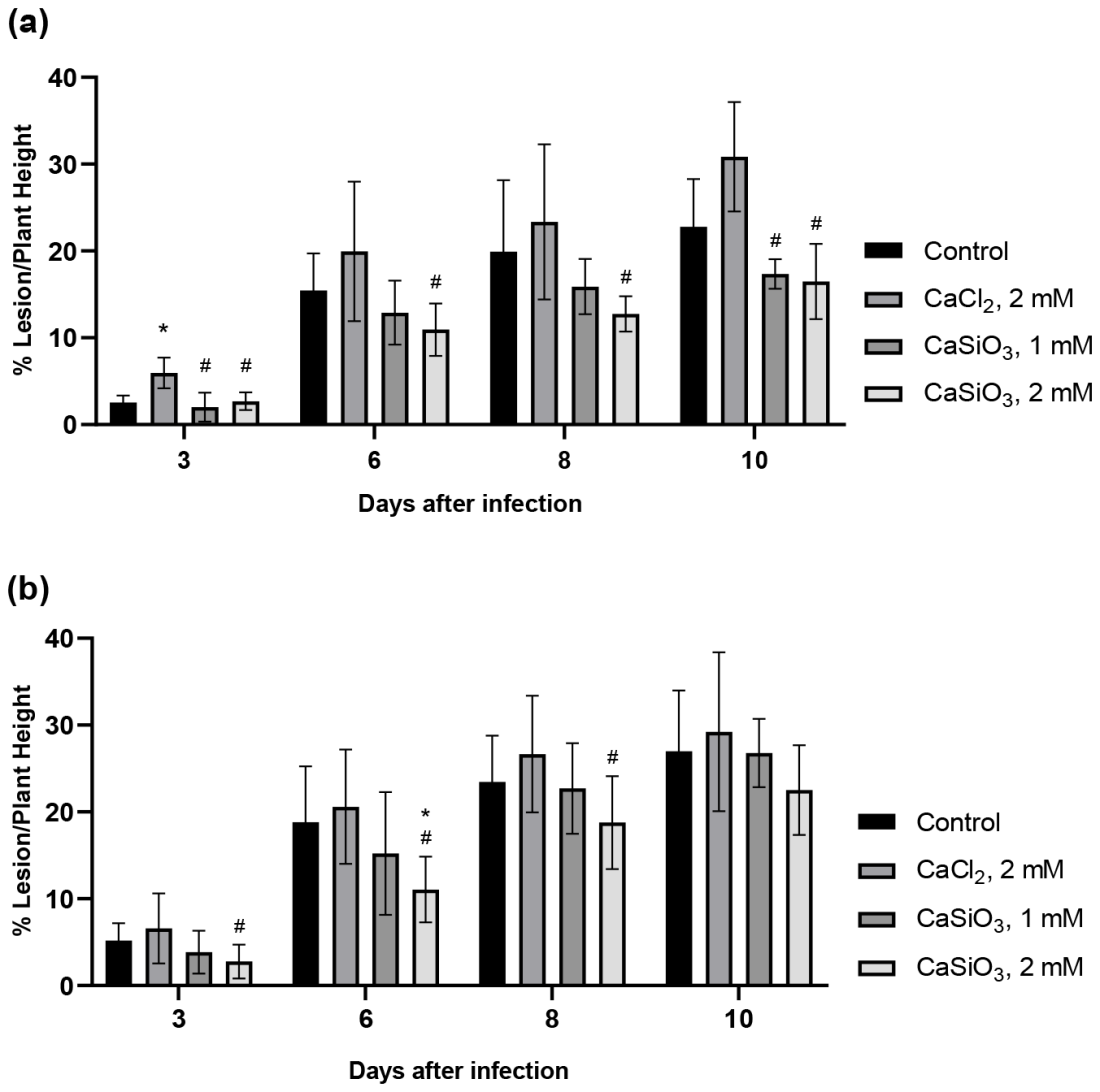
Lesion in the stem of genotype A **(a)** and genotype B **(b)** plants after inoculation with *Neopestalotiopsis lusitanica*. The results were expressed as means of percentage of plant lesions [(lesion size/plant height) x 100] ± SD. Statistically significant differences compared to control plants are represented with an asterisk, and statistically significant differences compared to the element control are represented with a cardinal (Sidak´s multiple comparison analysis, *P* < 0.05).

Regarding lesion size, in genotype A (**Figure 8a**), plants treated with 2 mM calcium chloride showed a marked increase at 3 days post-infection. In contrast, both calcium silicate treatments resulted in smaller lesion sizes compared to the control and element control. Over the entire evaluation period, calcium silicate treatments consistently reduced lesion size, with 2 mM calcium silicate showing the most pronounced effect. In genotype B (**Figure 8b**), calcium silicate treatments initially reduced lesion size relative to both the control and the element control, with 2 mM calcium silicate causing the greatest reduction at early time points. This trend was maintained through days 6 and 8, during which 2 mM calcium silicate significantly decreased lesion size compared to both controls. However, by day 10, differences among treatments were no longer significant. Despite this, 2 mM calcium silicate consistently maintained the lowest lesion percentage throughout the experiment.

**Figure 9:**
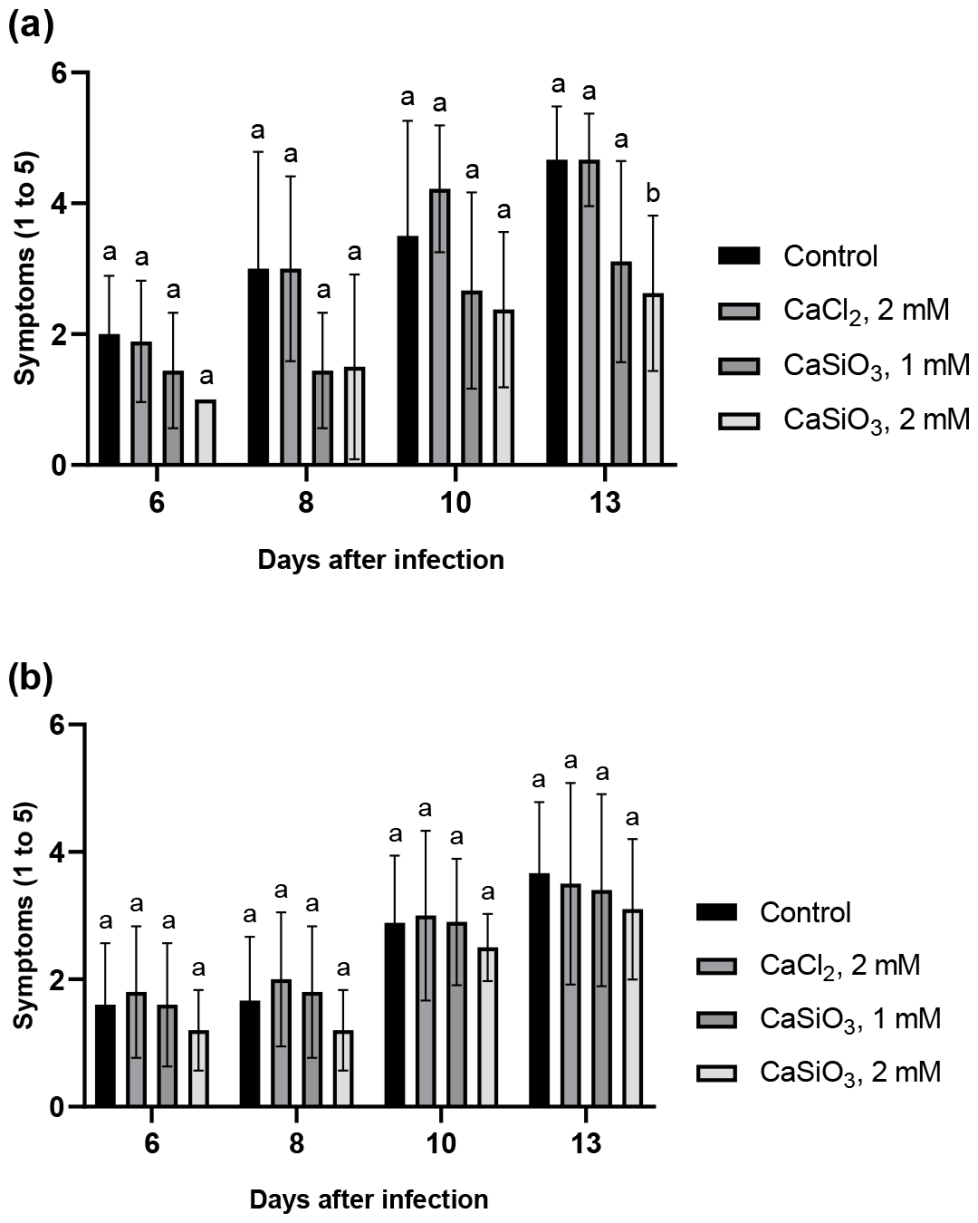
Qualitative analysis of the symptoms presented by plants of genotypes **(a)** and **(b)** after inoculation with *Neopestalotiopsis lusitanica*, according to the severity of symptoms (Level 1 – Necrotic lesions are present on the stem, but the plant shows no visible signs of physiological decline; Level 2 – Initial leaf desiccation is observed, though the plant remains overall healthy; Level 3 – Foliar desiccation is evident, especially at the apex, but the plant is still viable; Level 4 – Widespread desiccation and prominent necrotic lesions are present; the plant is severely affected and approaching death; Level 5 – The plant is fully desiccated and/or dead.). The results were expressed as the mean of symptoms (1 to 5) ± SD. Statistically significant differences are represented with different letters (Tukey test, *P* < 0.05).

Overall plant health was further assessed using a 5-level symptom scale (**Figure 9**) at 3, 5, 8, 10 and 13 days post-inoculation. In genotype A, no statistically significant differences were observed between days 6 and 10 post-infection. However, plants treated with calcium silicate generally showed lower symptom severity than both the control and element control groups. By day 13 post-infection, plants treated with 2 mM calcium silicate showed significantly reduced disease symptoms compared to control and element control. These findings further support the protective effects of this treatment, consistent with the observed trends in mortality rates and lesion size. In genotype B, no statistically significant differences were detected among treatments. Nonetheless, calcium silicate-treated plants consistently displayed lower symptom severity than the water and element controls, reinforcing the comparatively weaker effect of Si in this genotype.

### 3.4 Silicon absorption and mobility in the eucalyptus plant

**Figure 10:**
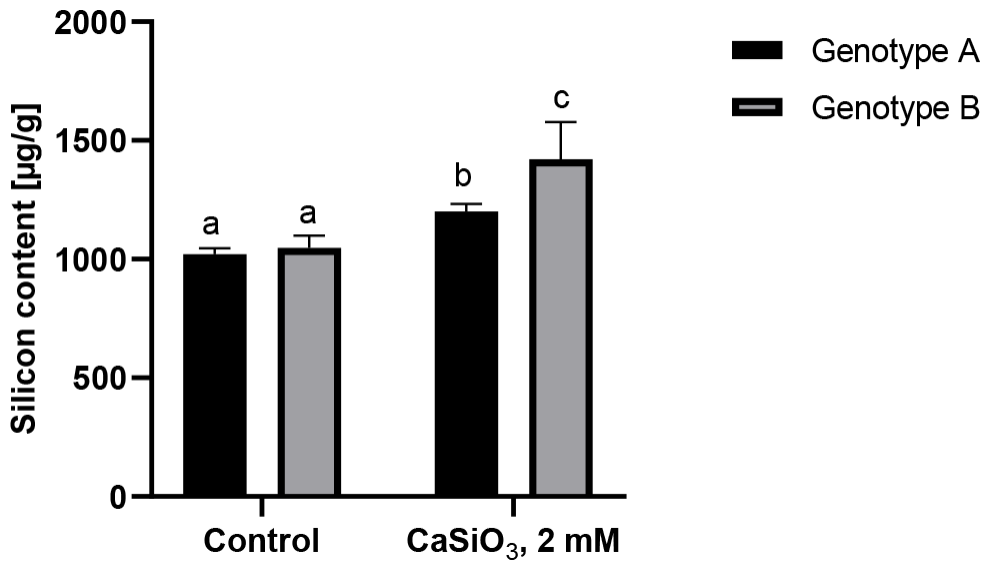
Silicon content in leaves of plants of genotype A and B. The results were expressed as the mean of Si content by dry mass ± SD. Statistically significant differences are represented with different letters (Tukey test, P < 0.05).

To assess Si absorption efficiency and mobility in *E. globulus*, Si content was determined in the leaves of both genotypes A and B, grown for 5 weeks under 2 mM calcium silicate treatment, and in the control plants. In **Figure 10**, we can see that plants of both genotypes treated with Si accumulated this element in their leaves, indicating that they absorbed and translocated it from the roots to the leaves. A considerably higher amount of Si was detected in the leaves of genotype B plants.

## 4 Discussion

In this study, we assessed the impact of Si on *E. globulus*, focusing on growth, drought resistance, and protection against *N. lusitanica* disease in two genotypes with differing susceptibility to this fungus. We first evaluated the effects of Si on plant growth and productivity-related parameters, focusing on biometric traits and physiological indicators, including photosynthetic pigments, sugar content, and starch levels. No evidence of phytotoxicity was observed, as neither plant mortality nor visible stress symptoms were detected in Si-treated plants. Although no significant increase in growth was observed in genotype A, plants of genotype B treated with 1 or 2 mM calcium silicate exhibited significantly greater growth compared with control plants. In contrast, studies with other *Eucalyptus* species, such as *E. camaldulensis* and *E. urophylla × E. grandis*, reported no differences in plant growth with Si application at various concentrations (Queiroz et al., 2018; Mateus et al., 2017). The increase in growth observed in genotype B was accompanied by significantly higher levels of photosynthetic pigment and starch, while sugar content remained unchanged across treatments, as expected due to tight regulation (Sakr et al., 2018). However, these effects appear to be genotype-dependent, as genotype A showed no significant increase in plant growth or pigment content despite a slight increase in starch levels. Similar improvements in photosynthesis, photosynthetic pigments, and starch levels following Si application have been reported in other eucalyptus species (Lima et al., 2016; Mateus et al., 2017; Junior et al., 2021). Although previous studies in other *Eucalyptus* species, as well as our results for *E. globulus* genotype A, showed no significant growth effects in response to Si application, this may not apply to all species or genotypes, as demonstrated by our findings for genotype B. The growth results obtained in this work are consistent with the silicon levels detected in the leaves of each genotype, with genotype B showing a greater capacity for silicon uptake and/or retranslocation.

Despite these findings, research on the effects of Si in eucalyptus remains limited. To our knowledge, no studies have specifically examined its impact on *E. globulus* growth, drought tolerance, or resistance to fungal infection. In addition to growth effects, we also analyzed impact of Si on drought stress in plants of genotype A. Our results show that Si alleviated drought stress induced by PEG-6000, with plants treated with 2 mM calcium silicate growing significantly more than the PEG-6000 control and reaching values comparable to those of unstressed plants, suggesting that Si counteracted drought stress in *E. globulus*. While not previously reported in *E. globulus* and rarely studied in eucalyptus and other woody species, Si’s role in drought resistance is well-documented in crops, although its underlying mechanisms remain unclear. Several authors have reported that Si improved crop growth under drought stress, mitigating its negative effects on growth and biomass. (Verma et al., 2020; Cheraghi et al., 2023, 2024; Silva et al., 2025). Si may reduce transpiration, enhance root growth, and support metabolism (Broadley et al., 2012; Luyckx et al., 2017; Tripathi et al., 2021; Nascimento-Silva, 2023). The protective effect of Si in eucalyptus observed in this study was reflected in reduced lipid peroxidation, as indicated by lower MDA content, confirming its role in protecting cellular membranes. Studies in different plant species have also shown that Si reduces MDA levels under drought stress (Verma et al., 2020; Xu et al., 2017; Malik et al., 2021). Furthermore, in our study, calcium silicate treatments significantly reduced Pro levels in PEG-treated plants, restoring them to levels comparable to those of unstressed plants. Proline, a key non-enzymatic component, plays a crucial role in maintaining cellular homeostasis and signaling. It mitigates oxidative damage by scavenging hydroxyl radicals and reducing H_2_O_2_ levels (Zandi and Schnug, 2022; Rudenko et al., 2023). Pro levels in *E. globulus* have been shown to increase under drought stress (Shvaleva et al., 2005) and studies in several crops have also reported that Si reduced Pro levels under drought stress (Xu et al., 2017; Parveen et al., 2019; Verma et al., 2020). Moreover, under drought stress, calcium silicate treatment significantly reduced *E. globulus* GSH levels relative to the control, restoring them to levels comparable to those of unstressed plants. Reduced glutathione (GSH) plays a crucial role in detoxifying reactive oxygen species (ROS) by neutralizing them through oxidation (Rai et al., 2023). This suggests that Si may help reduce oxidative stress in *E. globulus*. One possible explanation is that Si alleviated oxidative stress through multiple mechanisms, thereby reducing the need for high GSH levels. However, this contrasts with previous studies showing that Si typically increases GSH levels to enhance ROS scavenging (Malik et al., 2021). For example, Xu et al. (2017) and Shi et al. (2016) reported increased GSH levels under drought stress in wheat and tomato, respectively. Regarding H_2_O_2_, a signaling molecule that can trigger cell death at high concentrations (Zandi and Schnug, 2022; Mishra et al., 2023), no significant differences between stressed and unstressed *E. globulus* plants were observed. This suggests that 5% PEG-6000-induced stress was insufficient to trigger H_2_O_2_ production or that H_2_O_2_ is not a sensitive indicator of drought stress in this context. The enzymatic antioxidant system also mitigates cellular damage by scavenging H_2_O_2_ through CAT, APX, and GPOX. APX has a higher affinity for H_2_O_2_, while CAT has a faster turnover (Hasanuzzaman et al., 2020; Zandi and Schnug, 2022). GPOX removes excess H_2_O_2_ and contributes to lignin biosynthesis and stress defense (Zandi and Schnug, 2022). In addition, abiotic stress stimulates nitrogen-related enzymes, such as GOGAT and GDH, with GDH being associated with increased proline metabolism and drought adaptation in several plant species (Xu et al., 2023). Our results showed no significant differences in the activity of CAT, APX and GPOX; however, treatment with 2 mM calcium silicate significantly increased GDH activity under drought stress. Xu et al. (2023) similarly reported increased GDH activity in Si-treated strawberries under limited irrigation.

Many studies highlight the role of Si in alleviating biotic stress, although its mechanisms remain ambiguous. Si is believed to strengthen physical barriers against pathogen infections and enhance plant antioxidant metabolism, similar to its role in abiotic stress resistance (Fauteux et al., 2005; Ahammed and Yang, 2021; Song et al., 2021). Overall, in the present study, calcium silicate treatments significantly reduced plant mortality, lesion size, and disease symptoms caused by *N. lusitanica* in both *E. globulus* genotypes, with the strongest effects observed in the more susceptible genotype A at 2 mM. These results suggest that 2 mM calcium silicate effectively enhances tolerance to fungal diseases in susceptible genotypes, whereas its benefits may be less pronounced in inherently more resistant genotypes. This finding highlights the genotype-dependent nature of both disease resistance and Si-mediated protection in *E. globulus*. The contrasting responses observed between genotypes further emphasize the importance of genetic factors in determining resistance, in agreement with previous studies on the use of Si for fungal disease control. To our knowledge, no studies have investigated the Si against *Neopestalotiopsis* species. However, Carré-Missio et al. (2010) reported that 30 g L^-^¹ potassium silicate reduced the severity of *Pestalotia longisetula* in strawberries by 61%. Similarly, Cai et al. (2008) found that 2 mM potassium silicate in rice enhanced lignification, increased defense-related enzyme activity, and promoted leaf silicification, reducing blast disease severity by 45%. Moreover, Liang et al. (2005) demonstrated that Si application increased cucumber resistance to powdery mildew and that root-applied Si induced systemic acquired resistance. More recently, Xiao et al. (2022) reported that 75 mg L^-^¹ potassium silicate reduced hyphal length and density on powdery mildew strawberry leaves, thereby decreasing the infected leaf area.

In the present study, we also evaluated the ability of the two *E. globulus* genotypes to absorb, transport, and accumulate Si. Our findings show that both genotypes absorbed Si supplied through soil irrigation and accumulate it in leaves at levels ranging from 1,2 to 1,4 mg/g dry weight, depending on the genotype. These values support the classification of *E. globulus* as a non-accumulator species and are consistent with previous reports for *Eucalyptus* species (Queiroz et al., 2018; Deshmukh et al., 2020). However, Si translocation appears to be genotype-dependent, with genotype B translocating more Si to the leaves than genotype A. This difference may explain the superior growth performance observed in genotype B plants following calcium silicate treatment. Despite exhibiting lower Si uptake and/or retranslocation, genotype A did not show reduced growth under unstressed conditions. Nevertheless, the amount of Si accumulated was sufficient to enhance resistance to both biotic and abiotic stress.

## 5 Conclusion

This study provides new insights into the potential benefits of incorporating Si into *E. globulus* nursery nutrition to enhance growth and resistance to biotic, such as *Neopestalotiopsis* sp, an emerging and increasingly important fungus in nursery production systems, and abiotic stresses, particularly drought stress. Si treatments, especially 2 mM calcium silicate, improved plant performance by promoting growth, increasing photosynthetic pigments and starch content, and enhancing drought tolerance by mitigating oxidative stress. Additionally, Si significantly reduced disease severity, delaying lesion formation and lowering plant mortality, highlighting its potential as a protective strategy against fungal pathogens in nursery-grown eucalyptus. From an applied perspective, these findings support the integration of silicon fertilization, particularly calcium silicate, into nursery management practices to improve plant quality and resilience before field transplantation. Although *E. globulus* can absorb and accumulate Si, it cannot be considered a typical Si-accumulator species, and its capacity to uptake and retranslocation appears to be genotype-dependent, influencing its physiological responses. Therefore, nursery management strategies should consider genotype-specific responses to optimize the benefits of Si fertilization. It is important to note that infection with *N. lusitanica* was artificially induced in this study. Future research should explore the application of calcium silicate treatment at a semi-operational scale in nurseries, under natural infection conditions and across different stages of plant production. Further studies should also investigate the response of calcium silicate-treated plants to combined biotic and abiotic stresses, which more accurately reflects the environmental conditions expected under ongoing climate change.

## Data Availability

The raw data supporting the conclusions of this article will be made available by the authors, without undue reservation.

## References

[B1] Ahammed G. J. Yang Y. (2021). Mechanisms of silicon-induced fungal disease resistance in plants. Plant Physiol. Biochem. 165, 200–206. doi: 10.1016/j.plaphy.2021.05.031 34052681

[B2] Ahmed A. H. H. Harb E. M. Higazy M. A. Morgan S. H. (2008). Effect of silicon and boron foliar applications on wheat plants grown under saline soil conditions. Int. J. Agric. Res. 3, 1–26. doi: 10.3923/ijar.2008.1.26

[B3] Artyszak A. (2018). Effect of silicon fertilization on crop yield quantity and quality—a literature review in Europe. Plants 7, 54. doi: 10.3390/plants7030054 29986422 PMC6161276

[B4] Bakhat H. F. Bibi N. Zia Z. Abbas S. Hammad H. M. Fahad S. . (2018). Silicon mitigates biotic stresses in crop plants: a review. Crop Prot. 104, 21–34. doi: 10.1016/j.cropro.2017.10.008 38826717

[B5] Barradas C. Phillips A. J. L. Correia A. Diogo E. Bragança H. Alves A. (2016). Diversity and potential impact of Botryosphaeriaceae species associated with Eucalyptus globulus plantations in Portugal. Eur. J. Plant Pathol. 146, 245–257. doi: 10.1007/s10658-016-0910-1 30311153

[B6] Basirat M. Mousavi S. M. Abbaszadeh S. Ebrahimi M. Zarebanadkouki M. (2019). The rhizosheath: a potential root trait helping plants to tolerate drought stress. Plant Soil. 445, 565–575. doi: 10.1007/s11104-019-04334-0 30311153

[B7] Belisário R. Aucique Pérez C. E. Abreu L. M. Salcedo S. S. Oliveira W. M. D. Furtado G. Q. (2020). Infection by Neopestalotiopsis spp. occurs on unwounded eucalyptus leaves and is favoured by long periods of leaf wetness. Plant Pathol. 69, 194–204. doi: 10.1111/ppa.13132 40046247

[B8] Bradford M. M. (1976). A rapid and sensitive method for the quantitation of microgram quantities of protein utilizing the principle of protein dye binding. Anal. Biochem. 72, 248–254. doi: 10.1006/abio.1976.9999 942051

[B9] Bragança H. Diogo E. L. F. Neves L. Valente C. Araújo C. Bonifácio L. . (2016). Quambalaria eucalypti, a pathogen of Eucalyptus globulus newly reported in Portugal and in Europe. For. Pathol. 46, 57–75. doi: 10.1111/efp.12221 40046247

[B10] Brito Â. Rocha M. Kaštovský J. Vieira J. Vieira C. P. Ramos V. . (2022). A new cyanobacterial species with a protective effect on lettuce grown under salinity stress: envisaging sustainable agriculture practices. J. Appl. Phycol. 34, 915–928. doi: 10.1007/s10811-022-02692-4 30311153

[B11] Broadley M. Brown P. Cakmak I. Ma J. F. Rengel Z. Zhao F. (2012). “ Chapter 8 – beneficial elements,” in Marschner’s Mineral Nutrition of Higher Plants, 3rd edn. Ed. Marschner P. ( Academic Press). doi: 10.1016/B978-0-12-384905-2.00008-X

[B12] Cai K. Gao D. Luo S. Zeng R. Yang J. Zhu X. (2008). Physiological and cytological mechanisms of silicon induced resistance in rice against blast disease. Physiol. Plant 134, 324–333. doi: 10.1111/j.1399-3054.2008.01140.x 18513376

[B13] Carré Missio V. Rodrigues F. A. Schurt D. A. Rezende D. C. Ribeiro N. B. Zambolim L. (2010). Aplicação foliar de silicato de potássio, acibenzolar S metil e fungicidas na redução da mancha de Pestalotia em morango. Trop. Plant Pathol. 35, 182–185. doi: 10.1590/S1982-56762010000300008 41099703

[B14] Cheraghi M. Motesharezadeh B. Mousavi S. M. Basirat M. Alikhani H. A. Zarebanadkouki M. (2024). Application of silicon improves rhizosheath formation, morpho physiological and biochemical responses of wheat under drought stress. Plant Soil 503, 263–281. doi: 10.1007/s11104-024-06584-z 30311153

[B15] Cheraghi M. Motesharezadeh B. Mousavi S. M. Ma Q. Ahmadabadi Z. (2023). Silicon (Si): a regulator nutrient for optimum growth of wheat under salinity and drought stresses – a review. J. Plant Growth Regul. 42, 5354–5378. doi: 10.1007/s00344-023-10959-4 30311153

[B16] Correia B. Pintó Marijuan M. Neves L. Brossa R. Dias M. C. Costa A. . (2014). Water stress and recovery in the performance of two Eucalyptus globulus clones: physiological and biochemical profiles. Physiol. Plant 150, 580–592. doi: 10.1111/ppl.12110 24117924

[B17] Dasgupta M. G. Dharanishanthi V. (2017). Identification of PEG-induced water stress responsive transcripts using co-expression network in Eucalyptus grandis. Gene 627, 393–407. doi: 10.1016/j.gene.2017.06.050 28687330

[B18] Deshmukh R. Sonah H. Bélanger R. R. (2020). New evidence defining the evolutionary path of aquaporins regulating silicon uptake in land plants. J. Exp. Bot. 71, 6775–6788. doi: 10.1093/jxb/eraa342 32710120

[B19] Diogo E. Gonçalves C. I. Silva A. C. Valente C. Bragança H. Phillips A. J. L. (2021). Five new species of Neopestalotiopsis associated with diseased Eucalyptus spp. in Portugal. Mycol. Prog. 20, 1441–1456. doi: 10.1007/s11557-021-01741-5 30311153

[B20] Du B. Haensch R. Alfarraj S. Rennenberg H. (2024). Strategies of plants to overcome abiotic and biotic stresses. Biol. Rev. 99, 1524–1536. doi: 10.1111/brv.13079 38561998

[B21] FAO (1978). Eucalyptus globulus provenances by R.K. Orme. For. Genet. Resour. Inf. 7.

[B22] FAO (2001). Mean annual volume increment of selected industrial forest plantation species by L. Ugalde & O. Pérez. For. Plantation Thematic Pap. 1.

[B23] Fauteux F. Rémus-Borel W. Menzies J. G. Bélanger R. R. (2005). Silicon and plant disease resistance against pathogenic fungi. FEMS Microbiol. Lett. 249, 1–6. doi: 10.1016/j.femsle.2005.06.034 16006059

[B24] Ferreira S. Moreira E. Amorim I. Santos C. Melo P. (2019). Arabidopsis thaliana mutants devoid of chloroplast glutamine synthetase (GS2) have non-lethal phenotype under photorespiratory conditions. Plant Physiol. Biochem. 144, 365–374. doi: 10.1016/j.plaphy.2019.10.009 31622939

[B25] Frew A. Weston L. A. Reynolds O. L. Gurr G. M. (2018). The role of silicon in plant biology: a paradigm shift in research approach. Ann. Bot. 121, 1265–1273. doi: 10.1093/aob/mcy009 29438453 PMC6007437

[B26] Gaire S. Muzhinji N. Louws F. J. Adhikari T. B. (2026). Neopestalotiopsis spp. an invasive fungal pathogen, is a major threat to strawberry production: the current status, challenges, and future directions. Front. Plant Sci. 17, 1725321. doi: 10.3389/fpls.2026.1725321 41684855 PMC12891159

[B27] Guntzer F. Keller C. Meunier J. D. (2012). Benefits of plant silicon for crops: a review. Agron. Sustain. Dev. 31, 101–213. doi: 10.1007/s13593-011-0039-8 30311153

[B28] Hasanuzzaman M. Bhuyan M. H. M. B. Zulfiqar F. Raza A. Mohsin S. M. Mahmud J. A. . (2020). Reactive oxygen species and antioxidant defense in plants under abiotic stress: revisiting the crucial role of a universal defense regulator. Antioxidants 9, 681. doi: 10.3390/antiox9080681 32751256 PMC7465626

[B29] Junior J. P. D. S. Prado R. D. M. Morais T. C. B. D. Frazão J. J. Sarah M. M. D. S. Oliveira K. R. D. . (2021). Silicon fertigation and salicylic acid foliar spraying mitigate ammonium deficiency and toxicity in Eucalyptus spp. clonal seedlings. PloS One 16, e0250436. doi: 10.1371/journal.pone.0250436 33886651 PMC8061938

[B30] Khan A. A. Wang Y.-F. Akbar R. Alhoqail W. A. (2025). Mechanistic insights and future perspectives of drought stress management in staple crops. Front. Plant Sci. 16, 1547452. doi: 10.3389/fpls.2025.1547452 40212871 PMC11982952

[B31] Khan A. L. (2025). Silicon: a valuable soil element for improving plant growth and CO_2_ sequestration. J. Adv. Res. 71, 43–54. doi: 10.1016/j.jare.2024.05.027 38806098 PMC12126704

[B32] Khan I. Awan S. A. Rizwan M. Brestic M. Xie W. (2023). Silicon: an essential element for plant nutrition and phytohormone signaling mechanism under stressful conditions. Plant Growth Regul. 100, 301–319. doi: 10.1007/s10725-022-00872-3 30311153

[B33] Liang Y. C. Sun W. C. Si J. Römheld V. (2005). Effects of foliar- and root-applied silicon on the enhancement of induced resistance to powdery mildew in Cucumis sativus. Plant Pathol. 54, 678–685. doi: 10.1111/j.1365-3059.2005.01246.x 40046247

[B34] Lichtenthaler H. K. Buschmann C. (2001). Chlorophylls and carotenoids: measurement and characterization by UV-VIS spectroscopy. Curr. Protoc. Food. Anal. Chem. 1, F4.3.1–F4.3.8. doi: 10.1002/0471142913.faf0403s01 41531421

[B35] Lima M. D. R. Barros U. O. Jr. Barbosa M. A. M. Segura F. R. Silva F. F. Batista B. L. . (2016). Silicon mitigates oxidative stress and has positive effects in Eucalyptus platyphylla under aluminium toxicity. Plant Soil Environ. 62, 164–170. doi: 10.17221/85/2016-PSE 42463996

[B36] Luyckx M. Hausman J.-F. Lutts S. Guerriero G. (2017). Silicon and plants: current knowledge and technological perspectives. Front. Plant Sci. 8, 411. doi: 10.3389/fpls.2017.00411 28386269 PMC5362598

[B37] Ma J. F. Yamaji N. Tamai K. Mitani N. (2007). Genotypic difference in silicon uptake and expression of silicon transporter genes in rice. Plant Physiol. 145, 919–924. doi: 10.1104/pp.107.107599 17905867 PMC2048782

[B38] Maharachchikumbura S. S. N. Hyde K. D. Groenewald J. Z. Xu J. Crous P. W. (2014). Pestalotiopsis revisited. Stud. Mycol. 79, 121–186. doi: 10.1016/j.simyco.2014.09.005 25492988 PMC4255583

[B39] Malik M. A. Wani A. H. Mir S. H. Rehman I. U. Tahir I. Ahmad P. . (2021). Elucidating the role of silicon in drought stress tolerance in plants. Plant Physiol. Biochem. 165, 187–195. doi: 10.1016/j.plaphy.2021.04.021 34049031

[B40] Mariz-Ponte N. Mendes R. J. Sario S. Oliveira J. M. P. F. Melo P. Santos C. (2018). Tomato plants use non-enzymatic antioxidant pathways to cope with moderate UV-A/B irradiation: a contribution to the use of UV-A/B in horticulture. J. Plant Physiol. 211, 32–42. doi: 10.1016/j.jplph.2017.11.013 29223880

[B41] Mateus N. S. Ferreira E. V. O. Toledo F. H. S. F. Lavres J. Gonçalves J. L. M. (2017). Do the nutrition and physiology of eucalyptus seedlings respond to silicon (Si) supply? Aust. J. Crop Sci. 11, 1086–1093. doi: 10.21475/ajcs.17.11.09.pne422

[B42] Mishra N. Jiang C. Chen L. Paul A. Chatterjee A. Shen G. (2023). Achieving abiotic stress tolerance in plants through antioxidative defense mechanisms. Front. Plant Sci. 14, 1110622. doi: 10.3389/fpls.2023.1110622 37332720 PMC10272748

[B43] Nascimento-Silva K. (2023). Influence of silicon on tolerance to water deficit of peach trees. HortScience 58, 449–452. doi: 10.21273/HORTSCI16881-22

[B44] Pandey R. Singh C. Mishra S. Abdulraheem M. I. Vyas D. (2025). Silicon uptake and transport mechanisms in plants: processes, applications and challenges in sustainable plant management. Biol. Futura 76, 19–31. doi: 10.1007/s42977-024-00247-x 39587007

[B45] Parmar H. Goel A. Gelaw T. A. Reddy M. K. (2025). Enhancing drought resilience in crops: mechanistic approaches in the face of climate challenge. Plant Mol. Biol. 115, 82. doi: 10.1007/s11103-025-01616-3 40622492

[B46] Parveen A. Liu W. Hussain S. Asghar J. Perveen S. Xiong Y. (2019). Silicon priming regulates morpho-physiological growth and oxidative metabolism in maize under drought stress. Plants 8, 431. doi: 10.3390/plants8100431 31635179 PMC6843370

[B47] Pilon C. Soratto R. P. Moreno L. A. (2013). Effects of soil and foliar application of soluble silicon on mineral nutrition, gas exchange, and growth of potato plants. Crop Sci. 53, 1605–1614. doi: 10.2135/cropsci2012.10.0580

[B48] Pirralho M. Flores D. Sousa V. B. Quilhó T. Knapic S. Pereira H. (2014). Evaluation on papermaking potential of nine Eucalyptus species based on wood anatomical features. Ind. Crops Prod. 54, 327–334. doi: 10.1016/j.indcrop.2014.01.040 38826717

[B49] Queiroz D. L. D. Camargo J. M. M. Dedecek R. A. Oliveira E. B. D. Zanol K. M. R. Melido R. C. N. (2018). Absorção e translocação de silício em mudas de Eucalyptus camaldulensis. Ciênc Florest 28, 632–640. doi: 10.5902/1980509832053

[B50] Rai G. K. Kumar P. Choudhary S. M. Singh H. Adab K. Kosser R. . (2023). Antioxidant potential of glutathione and crosstalk with phytohormones in enhancing abiotic stress tolerance in crop plants. Plants 12, 1133. doi: 10.3390/plants12051133 36903992 PMC10005112

[B51] Ranjan A. Sinha R. Bala M. Pareek A. Singla-Pareek S. L. Singh A. K. (2021). Silicon-mediated abiotic and biotic stress mitigation in plants: underlying mechanisms and potential for stress resilient agriculture. Plant Physiol. Biochem. 163, 15–25. doi: 10.1016/j.plaphy.2021.03.044 33799014

[B52] Roque J. Brito Â. Rocha M. Pissarra J. Nunes T. Bessa M. . (2023). Isolation and characterization of soil cyanobacteria and microalgae and evaluation of their potential as plant biostimulants. Plant Soil 493, 115–136. doi: 10.1007/s11104-023-06217-x 30311153

[B53] Rudenko N. N. Vetoshkina D. V. Marenkova T. V. Borisova-Mubarakshina M. M. (2023). Antioxidants of non-enzymatic nature: their function in higher plant cells and the ways of boosting their biosynthesis. Antioxidants 12, 2014. doi: 10.3390/antiox12112014 38001867 PMC10669185

[B54] Saadaoui E. Yahia K. B. Dhahri S. Jamaa M. L. B. Khouja M. L. (2017). An overview of adaptive responses to drought stress in Eucalyptus spp. For. Stud. 67, 86–96. doi: 10.1515/fsmu-2017-0014 31755547

[B55] Sakr S. Wang M. Dédaldéchamp F. Perez-Garcia M.-D. Ogé L. Hamama L. . (2018). The sugar-signaling hub: overview of regulators and interaction with the hormonal and metabolic network. Int. J. Mol. Sci. 19, 2506. doi: 10.3390/ijms19092506 30149541 PMC6165531

[B56] Santos G. S. Mafia R. G. Aguiar A. M. Zarpelon T. G. Damacena M. B. Barros A. F. . (2020). Stem rot of eucalyptus cuttings caused by Neopestalotiopsis spp. in Brazil. J. Phytopathol. 186, 311–321. doi: 10.1111/jph.12894 40046247

[B57] Shi Y. Zhang Y. Han W. Feng R. Hu Y. Guo J. . (2016). Silicon enhances water stress tolerance by improving root hydraulic conductance in Solanum lycopersicum L. Front. Plant Sci. 7, 196. doi: 10.3389/fpls.2016.00196 26941762 PMC4761792

[B58] Shi Y. Zhang Y. Yao H. Wu J. Sun H. Gong H. (2014). Silicon improves seed germination and alleviates oxidative stress of bud seedlings in tomato under water deficit stress. Plant Physiol. Biochem. 78, 27–36. doi: 10.1016/j.plaphy.2014.02.009 24607576

[B59] Shvaleva A. L. Silva F. C. E. Breia E. Jouve L. Hausman J.-F. Almeida M. H. . (2005). Metabolic responses to water deficit in two Eucalyptus globulus clones with contrasting drought sensitivity. Tree Physiol. 26, 239–248. doi: 10.1093/treephys/26.2.239 16356921

[B60] Shwethakumari U. Prakash N. B. (2018). Effect of foliar application of silicic acid on soybean yield and seed quality under field conditions. J. Indian Soc Soil Sci. 66, 406–414. doi: 10.5958/0974-0228.2018.00051.8

[B61] Silva M. R. C. Diogo E. Bragança H. MaChado H. Phillips A. J. L. (2014). Teratosphaeria gauchensis associated with trunk, stem and foliar lesions of Eucalyptus globulus in Portugal. For. Pathol. 45, 224–234. doi: 10.1111/efp.12160 40046247

[B62] Silva L. F. S. Lima A. P. L. Lima S. F. Flach L. C. Blini L. P. Alves V. C. D. . (2025). Silicon and phytohormone-based biostimulant applied to eucalyptus seedlings under water stress conditions. Braz. J. Biol. 85, e298015. doi: 10.1590/1519-6984.298015 41221940

[B63] Silva M. C. MaChado H. N. Neves L. Araujo C. Phillips A. J. L. (2012). Mycosphaerella and Teratosphaeria species associated with Mycosphaerella leaf disease on Eucalyptus globulus in Portugal. For. Syst. 21, 300–305. doi: 10.5424/fs/2012212-02211 42463968

[B64] Song X. P. Verma K. K. Tian D. D. Zhang X. Q. Liang Y. J. Huang X. . (2021). Exploration of silicon functions to integrate with biotic stress tolerance and crop improvement. Biol. Res. 54, 19. doi: 10.1186/s40659-021-00344-4 34238380 PMC8265040

[B65] Tenguri P. Chander S. Ellur R. K. Yele Y. Sundaran A. P. Nagaraju M. T. . (2023). Effect of silicon application to the rice plants on feeding behaviour of the brown Planthopper, Nilaparvata lugens (Stål) under elevated CO2. Silicon 15, 5811–5820. doi: 10.1007/s12633-023-02480-w 30311153

[B66] Tomé M. Almeida M. H. Barreiro S. Branco M. R. Deus E. Pinto G. . (2021). Opportunities and challenges of Eucalyptus plantations in Europe: the Iberian Peninsula experience. Eur. J. For. Res. 140, 489–510. doi: 10.1007/s10342-021-01358-z 30311153

[B67] Tripathi D. K. Vishwakarma K. Singh V. P. Prakash V. Sharma S. Muneer S. . (2021). Silicon crosstalk with reactive oxygen species, phytohormones and other signaling molecules. J. Hazard. Mater. 408, 128420. doi: 10.1016/j.jhazmat.2020.124820 33516974

[B68] Verma K. K. Anas M. Chen Z. Rajput V. D. Malviya M. K. Verma C. L. . (2020). Silicon supply improves leaf gas exchange, antioxidant defense system and growth in Saccharum officinarum responsive to water limitation. Plants 9, 1032. doi: 10.3390/plants9081032 32823963 PMC7464948

[B69] Wang M. Gao L. Dong S. Sun Y. Shen Q. Guo S. (2017). Role of silicon on plant–pathogen interactions. Front. Plant Sci. 8, 701. doi: 10.3389/fpls.2017.00701 28529517 PMC5418358

[B70] Wang M. Wang R. Mur L. A. J. Ruan J. Shen Q. Guo S. (2021). Functions of silicon in plant drought stress responses. Hortic. Res. 8, 254. doi: 10.1038/s41438-021-00681-1 34848683 PMC8633297

[B71] White B. Tubana B. Babu T. Mascagni H. Agostinho F. Datnoff L. . (2017). Effect of silicate slag application on wheat grown under two nitrogen rates. Plants 6, 47. doi: 10.3390/plants6040047 29019922 PMC5750623

[B72] Wu Q. S. Wan X. Y. Su N. Cheng Z. J. Wang J. K. Lei C. L. . (2006). Genetic dissection of silicon uptake ability in rice (Oryza sativa L.). Plant Sci. 171, 441–448. doi: 10.1016/j.plantsci.2006.05.001 25193641

[B73] Xiao J. Li Y. Jeong B. R. (2022). Foliar silicon spray before summer cutting propagation enhances resistance to powdery mildew of daughter plants. Int. J. Mol. Sci. 23, 3803. doi: 10.3390/ijms23073803 35409165 PMC8998806

[B74] Xu L. Islam F. Ali B. Ghani M. A. Pei Z. Zhou W. (2017). Silicon and water-deficit stress differentially modulate physiology and ultrastructure in wheat (Triticum aestivum L.). 3 Biotech. 7, 273. doi: 10.1007/s13205-017-0904-5 28794928 PMC5538996

[B75] Xu X. Zou G. Li Y. Sun Y. Liu F. (2023). Silicon application improves strawberry plant antioxidation ability and fruit nutrition under both full and deficit irrigation. Sci. Hortic. 309, 111689. doi: 10.1016/j.scienta.2022.111684 38826717

[B76] Yang L. Kong J. Gao Y. Chen Z. Lin Y. Zeng S. . (2023). A simulated drier climate reduces growth and alters functional traits of Eucalyptus trees: a three-year experiment in South China. For. Ecol. Manag 549, 121435. doi: 10.1016/j.foreco.2023.121435 38826717

[B77] Zandi P. Schnug E. (2022). Reactive oxygen species, antioxidant responses and implications from a microbial modulation perspective. Biology 11, 155. doi: 10.3390/biology11020155 35205022 PMC8869449

