# Supplementary material for: The influence of silicon on growth, drought stress tolerance and defense against Neopestalotiopsis lusitanica, in Eucalyptus globulus
Source: Front Plant Sci. 2026 Aug 3;17:1894505. doi: 10.3389/fpls.2026.1894505 (PMC13478257; doi:10.3389/fpls.2026.1894505)
Supplement: Supplementary file 1 [file DataSheet1.docx]

Supplementary Material

**The influence of silicon on growth, water stress tolerance and defense against *Neopestalotiopsis* *lusitanica*, in *Eucalyptus globulus***

# 1 Supplementary Data

Tabel S1 - Composition of the water used in all experiments, sourced from an artisanal well located at RAIZ.

| Parameter | Result | Unit |
| --- | --- | --- |
| pH (at 20ºC) | 6.7 | Sörensen Scale |
| Conductivity (at 25ºC) | 166 | μS/cm |
| Total Suspended Solids (TSS) | < QL | mg/L |
| Carbonates, CO_3_^2-^ | < QL | mg/L |
| Bicarbonates, HCO_3_ | 41 | mg/L |
| Phosphorus, P | 0.12 | mg/L |
| Calcium, Ca | 4.2 | mg/L |
| Magnesium, Mg | 2.6 | mg/L |
| Sodium, Na | 21 | mg/L |
| Sodium Adsorption Ratio (SAR) | 2.0 |  |
| Potassium, K | 10 | mg/L |
| Iron, Fe | 0.13 | mg/L |
| Manganese, Mn | < QL | mg/L |
| Boro, B | 0.15 | mg/L |
| Coper, Cu | < QL | mg/L |
| Zinc, Zn | 0.38 | mg/L |
| Molybdenum, Mo | < QL | mg/L |
| Aluminium, Al | < QL | mg/L |
| Chlorides, Cl^-^ | 18 | mg Cl^-^/L |
| Nitrates, NO_3_^-^ | 0.090 | mg NO_3_^-^/L |
| Sulphates, SO_4_^2-^ | 15 | mg SO_4_^2-^/L |
| Chromium, Cr | < QL | mg/L |
| Ammoniacal Nitrogen | 0.17 | mg N/L |
| Silica, Si | 5.6 | mg/L |

Legend: QL - Quantification Limit

Figure S1- Illustration of *Neopestalotiopsis* inoculation process, showing lesion formation (brown areas surrounding the infection sites).

Figure S2 – Representative examples of disease symptoms caused by *Neopestalotiopsis*, classified into five categories. 1 – No symptoms, except for lesion formation on the stem; 2 – The apex (2a) or the leaves (2b) have started to dry, but the plant remains healthy and alive; 3 – Both the apex and leaves are drying, although the plant is still alive; 4 – Most of the plant is dry, lesions are extensive, and the plant is close to death; 5 – The plant is completely dead and dry.
